# Crowned Dens Syndrome: A Case Report Describing Refractory Symptoms

**DOI:** 10.1155/crrh/9005573

**Published:** 2026-08-28

**Authors:** Maha Rauf, Victor Y. Kuo, Elaine Cheung, Vishnu Bharani, Samuel Lai

**Affiliations:** ^1^ Department of Internal Medicine, University of California, Irvine, Orange, California, USA, berkeley.edu

## Abstract

Crowned dens syndrome (CDS) is a type of calcium pyrophosphate deposition disease that causes inflammation and pain due to calcium deposition in the odontoid process. Patients typically present with acute cervical pain often associated with fevers, elevated ESR and CRP, and leukocytosis. This presentation has a broad differential and raises concern for diagnoses such as giant cell arteritis and meningitis. In this case report, we describe a patient with classic CT scan findings of odontoid calcifications diagnosed with treatment‐refractory CDS.

## 1. Introduction

Calcium pyrophosphate deposition (CPPD) disease is a crystal arthropathy caused by calcium pyrophosphate (CPP) crystal deposition within joints, causing inflammation [1]. Crowned dens syndrome (CDS) is a type of CPPD involving the odontoid process [2]. Patients typically present with severe pain over the upper neck, sometimes radiating to the shoulder, temporal region, or occiput, limited neck rotation, elevated inflammatory markers, and sometimes fever. The duration of symptoms is variable, lasting from days to weeks [2, 3]. This nonspecific presentation lends itself to broad differential diagnoses including polymyalgia rheumatica, giant cell arteritis (GCA), spondylitis, or meningitis.

## 2. Case Presentation

The patient is a 74‐year‐old woman with a history of coronary artery bypass surgery, Type 2 diabetes, osteoarthritis (OA), and spinal stenosis who was admitted for evaluation of subacute headache and neck pain with new right hand/wrist swelling. The patient presented with 1 week of generalized weakness, impaired balance, neck pain, and stiffness. Additional symptoms included headache after onset of neck pain, jaw pain not triggered by chewing, low back pain, subjective fevers, new right‐hand swelling with pain and erythema, constipation, and dysuria. Patient denied radicular symptoms of her upper or lower extremities. She was seen twice at a community hospital 1 week prior to admission and was found to have a pseudomonal urinary tract infection (UTI), which was treated with Zosyn. At that time, her weakness was attributed to the UTI, and her headache and neck pain were attributed to a mechanical fall.

On initial evaluation, her vitals were within normal limits. Her physical exam was notable for markedly limited cervical rotation (more so than flexion and extension), no meningeal signs, abdominal tenderness localized to the lower quadrants, paraspinal muscle tenderness, soft tissue swelling of the right hand with tenderness over the dorsal aspect, intact peripheral pulses bilaterally, and no peripheral edema. Admission labs were notable for the following: serum sodium 131 mmol/L, white blood cell count 13.8 × 10^3^/μL (neutrophil predominant), hemoglobin 12 g/dL, C‐reactive protein (CRP) 26.9 mg/dL, and erythrocyte sedimentation rate (ESR) 118 mm/h.

**FIGURE 1:**
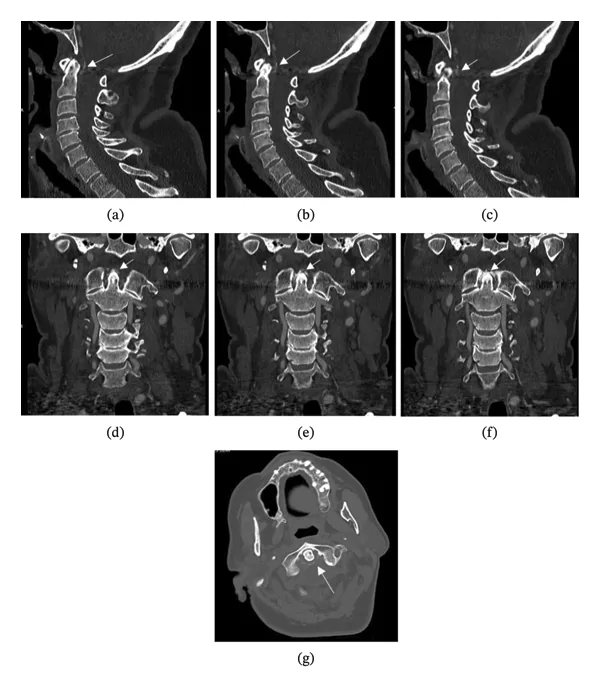
CT demonstrating calcification along the transverse ligament of the odontoid process. (a–c) Sagittal view. (d–f) Coronal view. (g) Axial view.

**FIGURE 2:**
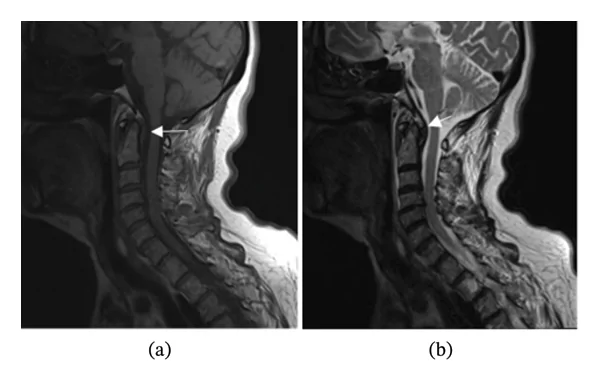
MRI demonstrating calcification along the transverse ligament of the odontoid process. (a) T1 weighted. (b) T2 weighted.

Workup initially included imaging (CT C‐spine; CT angiogram head/neck; MRI cervical/lumbar spine; and X‐rays of bilateral wrists, hands, and knees), labs (elevated CRP and ESR), and temporal artery biopsy. Cervical spine imaging revealed calcifications around the odontoid process along the transverse ligament concerning for CDS, mild degenerative changes, multilevel high‐grade cervical neuroforaminal stenosis, C4‐5 cord compression with intact cord signal, diffuse cervical interspinous and posterior paraspinal muscular edema, and asymmetric widening of the left atlantooccipital and atlantodens interval (Figures 1 and 2). X‐rays revealed soft tissue swelling of the right wrist with severe osteoarthrosis of the first carpometacarpal joint with heterotopic ossification, without bony erosion or periosteal reaction.

Rheumatology was consulted and recommended Celebrex 200 mg daily and colchicine 0.6 mg daily. Joint aspiration of the right wrist was performed with only minimal serosanguineous fluid obtained that was negative for crystals and insufficient fluid for cell count. Because her ESR and CRP did not initially improve following NSAIDs and colchicine, ophthalmology was consulted for temporal artery biopsy to rule out GCA, which was negative. Temporal artery ultrasound was also unremarkable. Orthopedic Spine was consulted for cervical myelopathy and neuroforaminal stenosis and felt the patient’s constellation of symptoms was more likely due to inflammatory arthritis. Other notable lab findings included slight positivity of rheumatoid factor and negative anticyclic citrullinated peptide antibodies. The patient’s symptoms were most consistent with CPPD with CDS. The patient was started on IV methylprednisolone with improvement in patient’s neck pain and mobility and decreased frequency of headaches. The patient continued to have milder symptoms, more prominently in the mornings. CRP additionally decreased to 11.6 mg/dL. Eventually, she was discharged with a prednisone taper and a colchicine regimen with plans for outpatient rheumatology follow‐up.

One week after discharge, she was readmitted for hyponatremia, at which time the patient noted mild improvement in her neck pain and headaches with adherence to prednisone and colchicine. Her ESR and CRP were increased from levels at previous discharge. Rheumatology was reengaged and recommended lumbar puncture given leukocytosis and persistent neck pain despite adequate therapy for CPPD and CDS. Cerebrospinal fluid studies showed low suspicion for infection or malignancy. The remainder of her infectious work‐up was also negative. A PET/CT scan was performed and did not show signs of large‐vessel vasculitis or malignancy. She was discharged on an increased regimen of colchicine 0.6 mg twice daily and a more gradual prednisone taper. The patient was subsequently seen by rheumatology in clinic with some improvement in symptoms and inflammatory markers. Given the refractory nature of her disease, the patient was initially considered for anakinra. However, she was not started on it given a positive hepatitis C antibody. The patient was ultimately started on chronic low‐dose prednisone as well as leflunomide and methotrexate, which led to resolution of her symptoms and elevated inflammatory markers over several months. Repeat imaging was not obtained given resolution of symptoms.

## 3. Discussion

CDS is a form of CPPD that has a clinical presentation that overlaps with several other conditions [4]. It primarily affects older patients and equally affects males and females. A recent meta‐analysis, including 196 patients with CDS, reported that only half of them had an initial correct diagnosis. This study found that 96.8% of patients had neck pain, 67.2% had limited range of motion, 43.6% had limited passive range of motion, 66.4% had temperatures over 38 C°, and 50.5% had leukocytosis. The patients who presented with restrictive passive/active range of motion or neck tenderness were significantly less likely to be initially misdiagnosed. The delay in initial diagnosis results in patients being put through invasive and/or expensive procedures such as lumbar punctures, temporal artery biopsy, or, in the case of our patient, a PET/CT scan in search of meningitis, malignancy, GCA, or polymyalgia rheumatica. This delay in diagnosis, along with these invasive procedures and imaging studies, places a financial strain on the healthcare system and can cause unnecessary stress for patients.

According to the 2023 ACR/EULAR classification criteria for CPPD disease, a diagnosis of CDS can be made with the presence of clinical symptoms along with classic imaging findings or CPP crystals in synovial fluid from a symptomatic joint. CT scan is the preferred imaging modality for diagnosis. Although several studies classify CDS as a rare cause of acute neck pain, more recent studies suggest it may be more common than previously recognized [5–8]. In a case series including 72 cases of CDS over 2 years across 8 hospitals in Japan found that the mean incidence of CDS was 4.6 ± 2.3 cases per year at each hospital. This study suggests that a patient’s inability to rotate their neck can be considered as a characteristic finding of CDS. Our patient presented with these characteristic findings; however, the concern of missing a more serious diagnosis such as meningitis or GCA remained and led to more invasive testing.

In this case report, we present a patient with classic symptomology and CT cervical spine findings for CPPD and CDS, including typical neck pain and stiffness with limited range of motion, elevated inflammatory markers, and characteristic calcifications of the transverse ligament of the dens on imaging. This patient had refractory disease that did not respond adequately to NSAIDs, colchicine, or prednisone, requiring chronic immunosuppression with methotrexate, leflunomide, and prednisone for resolution of symptoms and inflammation. A wide range of differential diagnoses were considered given the prolonged time course and refractory nature of the disease. Given the findings of neck pain and wrist pain, a variety of inflammatory arthritic diseases were considered, including psoriatic arthritis, rheumatoid arthritis, axial spondyloarthritis, systemic lupus erythematous, and adult‐onset Still’s disease, all of which were considered less likely given medical history, physical exam findings, lab results, and imaging findings as noted above. Polymyalgia rheumatica, fibromyalgia, and myositis were similarly unlikely. Given recent history of UTI, septic arthritis and cervical osteomyelitis and discitis were considered and ruled out by imaging and physical exam findings. With regard to the patient’s neck pain, headache, and systemic inflammation, GCA was ruled out with biopsy and imaging findings. Differential diagnoses for the neck pain alone were also considered, with meningitis and metastatic disease ruled out with lumbar puncture and imaging findings. It is possible that cervical muscle strain, myelopathy, and neuroforaminal stenosis contributed to the patient’s symptoms given the history of mechanical ground‐level fall and imaging findings. However, they are unlikely the primary cause given resolution with immunosuppressive therapies and the extended time course required for resolution. There were only mild degenerative changes of the cervical spine seen on imaging. Furthermore, it is unfortunate that the patient’s wrist arthrocentesis was nondiagnostic for CPPD or other etiologies of inflammatory arthritis. The patient did have signs of OA of the wrist, and given the acute nature of the patient’s presentation, it is possible the patient had an acute inflammatory arthritis superimposed on chronic wrist OA. Finally, calcifications of the transverse ligament can rarely also be seen with ossification of the transverse atlantal ligament (OTAL), although it is unlikely in this case. This patient did not have dense and thick ossification but did have systemic inflammation. The presentation was acute rather than insidious and progressive, and the patient eventually responded well to therapy. Overall, refractory CPPD with CDS remains the most likely diagnosis, with resolution after the patient was started on chronic immunosuppressive therapy.

Given that the incidence of CDS is higher than previously thought, increasing provider awareness of CDS as a cause of acute neck pain is important for earlier diagnosis and reduction of health care burden. In addition, increased reporting of the condition and its variable presentation will help streamline future research on the condition and improve the ease of diagnosis and treatment.

Underrecognized and often misdiagnosed, CDS can mimic other serious conditions. This case highlights CT imaging as a key diagnostic tool and the need for chronic immunosuppressive therapy in refractory cases where colchicine and NSAIDs have limited efficacy.

## Funding

No funding was received for this study.

## Conflicts of Interest

The authors declare no conflicts of interest.

## Data Availability

Data sharing is not applicable to this article as no datasets were generated or analyzed during the current study.
